# Supplementary material for: Discovery and application of insertion-deletion (INDEL) polymorphisms for QTL mapping of early life-history traits in Atlantic salmon
Source: BMC Genomics. 2010 Mar 8;11:156. doi: 10.1186/1471-2164-11-156 (PMC2838853; doi:10.1186/1471-2164-11-156)
Supplement: Additional file 2 — Information on developed 76 locus single-run INDEL panel in Atlantic salmon. Information on fluorescence labeling, primer concentrations, PCR pooling and links to alignments, INDEL motifs and GENESCAN (Burge and Karlin 1997) predictions of genes/exons are available in html format. [file 1471-2164-11-156-S2.ZIP › Additionalfile2/snpsummary20196.html]

```
Cluster 10740 Contig 1

prev  Summary    Contig List  next
```

Size of Consensus sequence = 1084

Number of sequences = 5

Minimum redundancy = 2

Key

A gi|117836406|gb|EG909102.1|EG909102 EST\_ssal\_evf\_11349 ssalevf mixed\_tissue Salmo salar cDNA Salmo salar cDNA clone ssal\_evf\_513\_233\_fwd 3', mRNA sequence  
B gi|75981151|gb|AM041446.1|AM041446 AM041446 pGemT-easy Atlantic salmon gill subtracted library (Aeromonas salmonicda infected salmon subtracted with uninfected) Salmo salar cDNA clone GT1D3, mRNA sequence  
C gi|117869732|gb|EG942428.1|EG942428 EST\_ssal\_evf\_25754 ssalevf mixed\_tissue Salmo salar cDNA Salmo salar cDNA clone ssal\_evf\_534\_039\_fwd 3', mRNA sequence  
D gi|117822770|gb|EG895466.1|EG895466 EST\_ssal\_evf\_4419 ssalevf mixed\_tissue Salmo salar cDNA Salmo salar cDNA clone ssal\_evf\_504\_085\_rev 5', mRNA sequence  
E gi|117869734|gb|EG942430.1|EG942430 EST\_ssal\_evf\_25755 ssalevf mixed\_tissue Salmo salar cDNA Salmo salar cDNA clone ssal\_evf\_534\_039\_rev 5', mRNA sequence

3 SNPs detected

A B C D E  cosegregation weighted

685 . T A T A   3/3 80.00
830 . - A - A   3/3 80.00
831 . - T - T   3/3 80.00
